# Metereological Table

**Published:** 1859-08

**Authors:** J. G. Westmoreland

**Affiliations:** Smithsonian Institution, July, 1859, at Atlanta, Ga.


					﻿S <	*	fc
___
§ o s >	. •	.	- •	• £: . I
’■S £	&	£
I £	04	«j «2	^z;
se .	j
"1 O 1
sW II	“	- —
1^ ?	3	•
<< j	02	“	“
j? £ I
,^1------------- ---------------------------------------------------I
~	a I	=«
K ?&J ;	pi 0®0©©©tOO©vOiOG OtO OOOOiOiOOiOUJiOtOOOOOOO t2
° " ■
C} .J I [ m	——	-----------------------------------
. O Q	S
Sf CO &	3	________ o
^ ?_	5 Ph ° ’" S, ~ ° ~ u~ ~ 2	1:7 ° ° °. ° ° ° 0 0 ‘° C> o 'O c"? 1C k-? O O 1O C O '“>
O 04	^2
o i ----------------------------------------------------------------s
'"2 *£i
S K>	3	__________ *
S,®	-r ’-' °©©-© t'JOOOtO © O WOOO OOiO O OO O uC tOOOtSOkC i
<zj |	t~-	•»
£ I	>
1-*“ ?x \---:_______________________________________________________t»
Zi	I	jz;	to 04	© 04	04	to	O to	>O	>O	o
• "	l—I	04 CO	LrJ !“!	<—1	04	to I-	04	I—	I
C5 2o	w	„	I
s „
w *	^gSS^SgE^'^gog^©©^©^©©^©^
•S	Eh	o  _	o
s’ <3 M- —----------------------------------------------------------
tbJ	g	*	§
”t5 II o <-:	oo © S So 2 ?° 2 22 ?°	2 2 2 2 22 ©	© 00 -*	© h* h <*>	© h §
O .	v-h A	^JL'-QU 1— CO I- O l^ I'- co o o o CO o O O O O CO CO OO CO CO CO CO CO CO 00 H
G I 5	04	5
gCb II g	,	-	-	j
•2 rT ij S •	51b2S2£3'!3^’^J3©©©H©M4©o4.4oco©TKo4'<*H#TiHoiH©o4oo® m
-S	EH	<	t'" I'I' -Oo o O tt O I-1-H H t-H I-I. H |. H [,	[,	t>.	«
g g j_______b- ___
£> j	u >o © to to >o ja to to to to © co to o o to to © o o to o o to co to © © «o to ©
<-O	’^^OrHQlQ'lrHOOr—i CM th © r-<OOOt—«OOOOOOOOOOOOO
r$ ’	Ai	o o o o’ o’ o’ o’ o’ o’ o’ o’ o o’ o o o o’ o o o o o o o o o o o o o o
1	_	c ~ O &> CO co CO CO CO CO CO C0 oo co co oi co co oo co co co co co co co co co co co co co
I w	_______ _	_____
*7?	M S	O O CO O O c ° 1£. 1O 0 LO O LO )-O o VO o O >O IO CC 1O >O cq lO O 1O O IO o
Jg	teH	Ol O1 rH H CO CO CM M 0 r-^ 04 Cl 04 rH T-I T-' rH r-, H O H O © TH t-1 T-< O o H rH rH
’JhL	5	ooooooooooo’oo’ooooooooo’oo’ooooooo
I p2»	C4 WCOCOCOCOCOCQCOQ4COCOCOCOCOCOCOCOCOCQCOCOCOCOCOCOCQC0COCOCOCQ
?	S I & ‘3	'J? l- O vd O O O © O LO O Ol 1O 10 O VO IO O O O IO O O O O to VO VO
g	W	,	l^r-J’-^OOMCOCMrHO^HOjOqrH^r-HOOrHOOOOOOr-HrHOOOrHO
< oooooooooooooooooooc^’ooo’oooo’oooo
t—	[	c,2 CO co co co co co co co co co co CO CO CO CO CO CO 04 co co co CO co co co co co co co
Day of Mo. |rHC^c^’^1xo«oi'*cooo»-ic<icoTti»Qot>.oocDOrHC^ co	i© «i> do o> o i-»
J	1	Hh!tHHt-<HHtHMH(NC1CMC1OIO4WC1C4(NC«3CQ
				

